# Cancer-predicting transcriptomic and epigenetic signatures revealed for ulcerative colitis in patient-derived epithelial organoids

**DOI:** 10.18632/oncotarget.25617

**Published:** 2018-06-19

**Authors:** Samaneh K. Sarvestani, Steven A. Signs, Veronique Lefebvre, Stephen Mack, Ying Ni, Andrew Morton, Ernest R. Chan, Xiaoxia Li, Paul Fox, Angela Ting, Matthew F. Kalady, Michael Cruise, Jean Ashburn, Jennifer Stiene, Wei Lai, David Liska, Shao Xiang, Emina H. Huang

**Affiliations:** ^1^ Department of Stem Cell Biology and Regenerative Medicine, Lerner Research Institute, Cleveland Clinic, Cleveland, Ohio, USA; ^2^ Department of Cell and Molecular Medicine, Cleveland Clinic, Cleveland, Ohio, USA; ^3^ Department of Pediatrics Baylor College of Medicine, Houston, Texas, USA; ^4^ Department of Genomic Medicine, Lerner Research Institute, Cleveland Clinic, Cleveland, Ohio, USA; ^5^ Department of Pathology, Case Western Reserve University, Cleveland, Ohio, USA; ^6^ Department of Epidemiology and Biostatistics, Institute for Computational Biology, Case Western Reserve University, Cleveland, Ohio, USA; ^7^ Department of Immunology, Lerner Research Institute, Cleveland Clinic, Cleveland, Ohio, USA; ^8^ Department of Colorectal Surgery, Cleveland Clinic, Cleveland, Ohio, USA; ^9^ Department of Pathology, Cleveland Clinic, Cleveland, Ohio, USA; ^10^ Department of Surgery, Wake Forest School of Medicine, Salem, North Carolina, USA

**Keywords:** ulcerative colitis, organoids, inflammation, H3K27Ac enhancer chromatin mark, colitis associated cancer

## Abstract

Ulcerative colitis (UC) is a prevalent form of inflammatory bowel disease (IBD) whose pathogenic mechanisms remain unclear. Elucidating these mechanisms is important to reduce UC symptoms and to prevent UC progression into colitis-associated colon cancer (CAC). Our goal was to develop and validate faithful, human-derived, UC models and analyze them at histologic, transcriptomic and epigenetic levels to allow mechanistic studies of UC and CAC pathogenesis.

We generated patient-derived primary-organoid cultures from UC and non-IBD colonic epithelium. We phenotyped them histologically and used next-generation-sequencing approaches to profile whole transcriptomes and epigenomes of organoids and primary tissues.

Tissue organization and expression of mucin 2 (MUC2) and lysozyme (LYZ) demonstrated histologic faithfulness of organoids to healthy and diseased colonic epithelium. Transcriptomic analyses showed increased expression of inflammatory pathways in UC patient-derived organoids and tissues. Profiling for active enhancers using the H3K27ac histone modification revealed UC-derived organoid enrichment for pathways indicative of gastrointestinal cancer, including S100 calcium-binding protein P (*S100P*), and revealed novel markers for GI cancer, including both *LYZ* and neuropeptide S receptor 1 (*NPSR1*). Immunolocalization showed increased levels of LYZ, S100P, and NPSR1 proteins in UC and CAC.

In conclusion, primary colonic organoid cultures from UC and non-IBD patients can be established that faithfully represent diseased or normal colonic states. These models reveal precancerous molecular pathways that are already activated in UC. The findings demonstrate the suitability of primary organoids for dissecting UC and CAC pathogenic mechanisms and suggest new targets for therapeutic intervention.

## INTRODUCTION

Inflammatory bowel disease (IBD) afflicts ∼1.4 million Americans and >2.5 million individuals worldwide [1]. Ulcerative colitis (UC) is one of two dominant IBD subtypes that include Crohn’s disease and UC. Clinical symptoms include bloody diarrhea, abdominal pain, malaise, and weight loss [1]. Under homeostatic colonic conditions, the colonic epithelium mediates nutrient absorption and secretion, and forms a physical wall segregating luminal contents from the underlying mucosa [2]. In UC, which is non-homeostatic, disruption of the barrier occurs due to imbalances of cell types and cell polarities. This results in access of luminal contents to the submucosa, and leads to exaggerated immune responses with recurring episodes of acute inflammation [1–4]. Colon removal is the only curative option when the chronic disease becomes refractory to standard medications. This operative procedure is also used to prevent progression of UC to colon-associated cancer (CAC). To date, the etiology of UC remains unclear. Genome-wide association studies have correlated UC with >160 susceptibility loci [5–8]. Some loci confer a predisposition toward disruption of the intestinal epithelial barrier, and others are relevant to epithelial restitution, microbial defense, innate immune regulation, reactive oxygen species generation, autophagy, and metabolic pathways [9]. Such genome-wide association studies thus allow identification of individuals at risk for developing UC. However, the mechanisms whereby these gene expression changes lead to UC remain largely unknown. When the mechanisms become known, they will enable the development of effective strategies to treat and cure the disease without removing the colon.

Current *in vivo* and *in vitro* animal models of IBD that exist in research typically involve genetic mutations and drugs, which do not reflect the human disease etiology. Patient-derived primary organoids, cultivated from biopsied colon specimens, not only surmount this obstacle, but also provide a multicellular model for studying IBD without the complexity of the gut microenvironment [10–12]. Organoids developed from human colon possess the stem cells (SCs) and the differentiated cell lineages that support absorption and secretion, with the three-dimensional cellular architecture of the large intestine, and thus constitute a refined model for studying epithelial homeostasis and its disruption in UC [13]. Recently, obstacles to the isolation and *in vitro* propagation of human three-dimensional cultures – with which to investigate the pathogenesis of IBD – have been conquered by Van Dussen [12]. However, the critical question of whether patient-derived organoids recapitulate the characteristics of primary UC tissues has not been answered. Answering this question was the first goal of this study.

Recent advances in understanding the genetics of common conditions, from blond hair [14], to ependymoma [15] and sporadic colorectal cancer (CRC) [16], have revealed epigenetic contributions to the phenotype of these states. While genetic susceptibility loci for UC may be known, the pathogenesis remains poorly understood. Profiling of active enhancers using the H3K27ac histone modification mark in UC organoids has not been reported and may therefore shed light on which of the susceptibility loci that may not only be correlated to disease states, but may also serve as functional or mechanistic targets for interventions. Thus, the second goal of our study was to use the next-generation-sequencing technologies of RNA sequencing (RNA-Seq) and epigenetic chromatin immunoprecipitation sequencing (ChIP-Seq) to investigate transcriptome and epigenetic signatures associated with epithelial organoids cultivated from colon tissues of UC patients.

Here we demonstrate that epithelial organoids derived from UC patients phenocopy the *in vivo* primary disease (i) not only at the histological level, but also at the (ii) transcriptomic and (iii) epigenetic levels. Further, we demonstrate that UC organoids and tissues already exhibit an oncogenic signature that we validate at each of these three levels using new markers.

## RESULTS

### Intestinal-epithelial organoids from UC patients histologically phenocopy their original primary tissues

Primary organoids were established using intestinal crypt units from the most inflamed distal portion of colons of UC patients and from the healthy colons of non-IBD patients (Supplementary Table 1). The full cohort included 10 non-IBD patients who had sporadic colon cancer (CRC), (60% male), and 10 who had UC (60% male). Tissues used to generate non-IBD organoids were generally retrieved from the farthest CRC tumor margin. The average age of patients with colitis who presented for operative resection was 42.3 ± 14.5 years; disease duration prior to presentation was 9.9 ± 9.7 years. The most common indication for surgical intervention was medical refractoriness. For non-IBD patients, age at presentation was 68.5 ± 7.6 years. Short tandem repeat (STR) analysis verified the unique patient origin of each organoid isolate (Supplementary Table 2). The epithelial lineage of each patient-derived organoid was confirmed by immunocytochemistry based on the presence of cytokeratin 19 (CK19) and absence of vimentin (VIM, Supplementary Figure 1).

**Figure 1:**
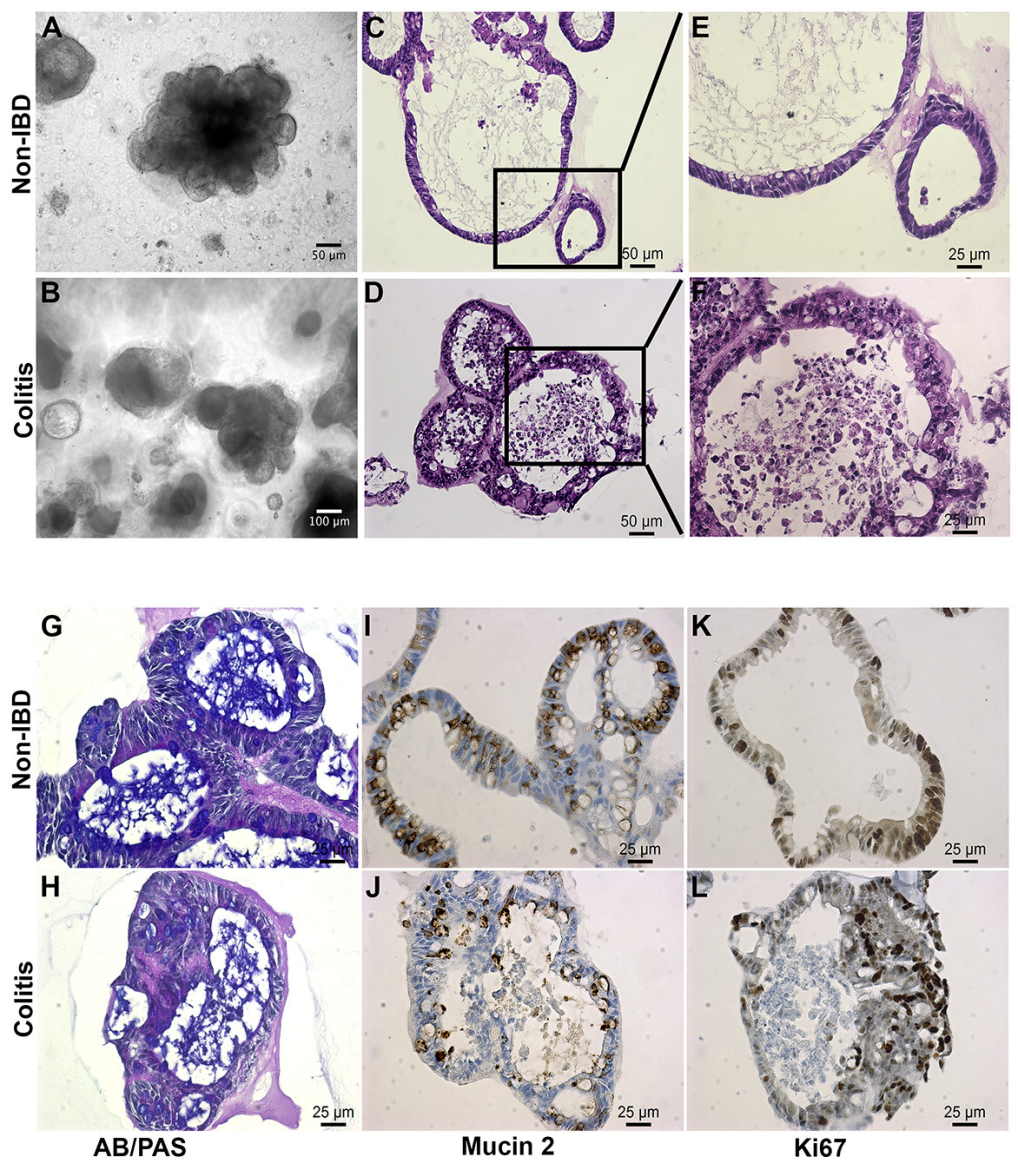
Colitic organoids phenocopy primary disease **(A, B)** Bright field images reveal polypoid 3D configurations with budding excrescences. **(C, E)** These images of embedded organoids document the relatively simple epithelium surrounding a cystic lumen containing mucus in the non-IBD organoid. **(D, F)** Contrast this epithelium with the more complex stratified epithelium from the colitis-derived organoid (n>20 each). **(G, H)** Alcian Blue (blue)/Periodic Acid Schiff (purple) staining is relatively weaker in colitis-derived organoids than in non-IBD organoids. **(I, J)** MUC2 level is reduced in colitic organoids relative to non-IBD derived organoids. **(K, L)** Ki67, a proliferation marker, is present in both non-IBD and colitic organoids. (n ≥ 3 per stain; Scale bars as shown).

Colon epithelial organoid isolated from non-IBD and UC patients showed similar histological morphology under low-magnification, bright-field microscopy (Figure 1A, 1B). After 3 weeks *in vitro* culture, organoids exhibited a characteristic three-dimensional gut-like architecture, including distinct crypt domains and an interior lumen.

Hematoxylin-eosin (H&E) staining of sections from non-IBD organoids revealed a well-organized, polarized and columnar epithelium, which is characteristic of healthy colonic mucosa (Figure 1C, 1E). In contrast, organoids from UC colon frequently presented with disorganized layers of a stratified epithelium, a characteristic of the epithelial lining of the large intestinal mucosa of patients with active UC (Figure 1D, 1F) [2].

The goblet cells of the intestinal mucosa produce a thick layer of membrane-associated mucins that protect the epithelial cell lining [17]. Staining of organoids with Alcian blue (AB) and periodic acid-Schiff (PAS) dyes revealed acidic and neutral mucus, respectively, consistent with the number of goblet cells. Non-IBD organoids possessed thick blankets of mucus, indicating normal goblet cell function (Figure 1G). Mucus layers in UC-derived organoids were often shallow and non-uniform, suggesting reduced mucin secretion (Figure 1H). This was supported by a two-fold decrease in MUC2-positive goblet-cell number in UC organoids (Figure 1I versus 1J; Supplementary Figure 1E). This is consistent with depletion of goblet cells and mucus layer in UC colon [2].

Immunohistochemical staining of organoid sections for the nuclear non-histone protein Ki-67 revealed uniform cellular proliferation throughout the columnar epithelium of non-IBD organoids (Figure 1K). Epithelial cells within UC organoids displayed a non-uniform pattern of proliferation, especially where expression was more concentrated in regions of disorganized stratified epithelium. This is consistent with accelerated epithelial cell turnover in areas of colonic mucosa undergoing remodeling in patients with active UC (Figure 1L, [1]).

Chromogranin A, a marker of enteroendocrine cells of the large intestine, was detected in limited but consistent numbers in both non-IBD and IBD organoids (Supplementary Figure 1F, 1G). Colonic enteroendocrine cells have been reported to exhibit both increased and decreased densities in UC [18]. Our findings regarding chromogranin A demonstrated consistency with the literature, proving fidelity of the model to primary tissues.

In conclusion, primary epithelial organoids generated from healthy, non-IBD colon specimens exhibited typical mini-gut architecture, and those generated from UC specimens presented with the distinct structural and cellular features of UC tissues [1]. The histological phenocopy of classical descriptions of disease, with larger numbers of primary colitic disease originating tissues lends credence to this model as a potential tool for revealing novel targets and mechanisms.

### Whole-transcriptome profiling reveals upregulation of inflammatory, metabolism, adhesion and oncogenesis pathways in UC relative to non-IBD organoids

**Figure 2:**
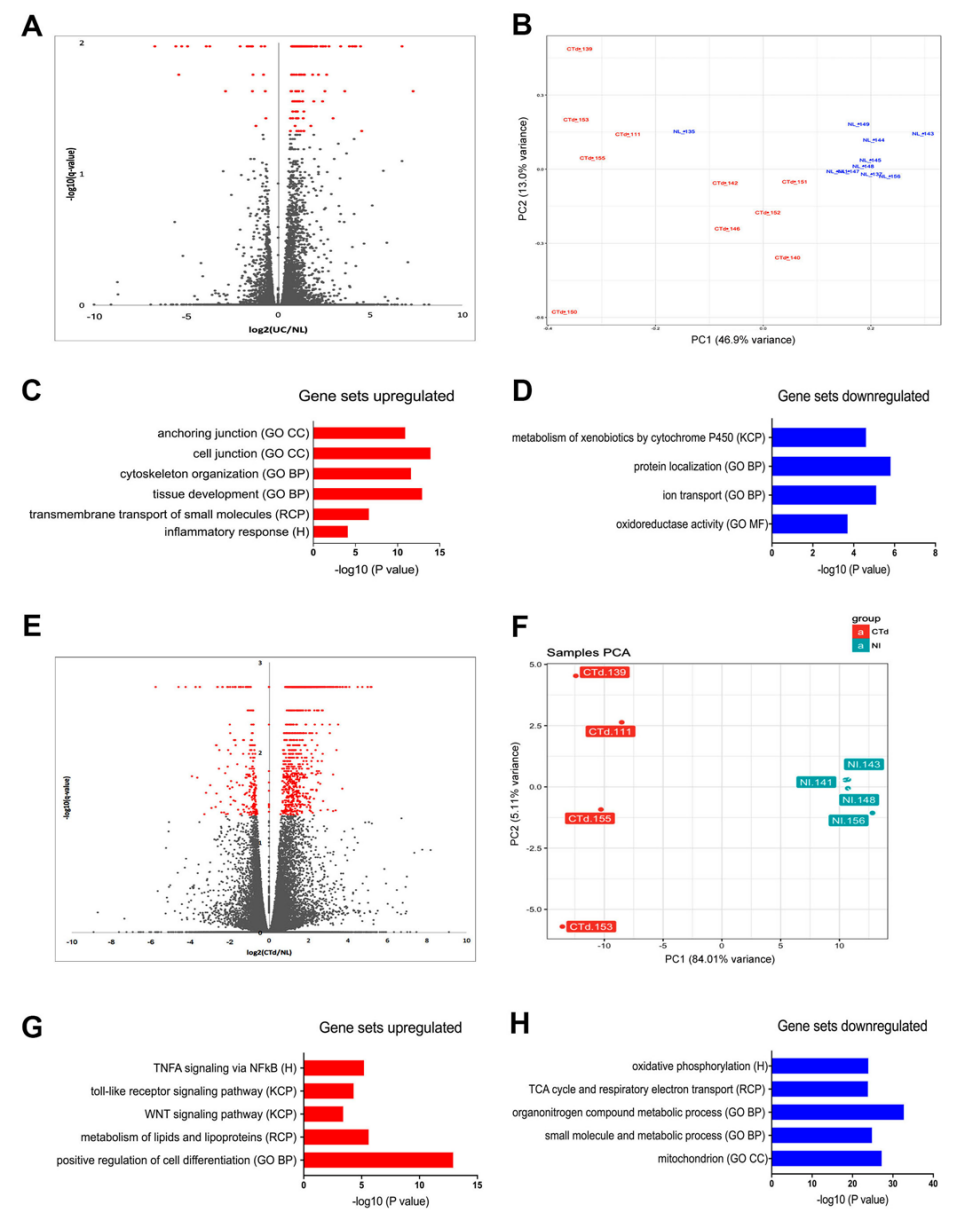
Transcriptome analysis of UC and non-IBD colonic organoids reveal distinct populations with clustering of gene set expression analyses consistent with colitic vs. non-IBD colon **(A)** Volcano plot of entire cohort indicates transcriptome differences based on the disease. FDR < 0.05 (Benjamini-Hochberg). Red - Colitis: Grey - non-IBD. **(B)** Principal component analysis of entire cohort reveals clustering of the populations of colitic organoids (red) versus non-IBD organoids (blue). **(C)** Enrichment of hits in Molecular Signatures Database (MSigDB) Gene Set Expression analysis of upregulated genes (colitis compared to non-IBD) reveals differential expression consistent with development and inflammation. Log 2-fold changes > 1.5, FDR p< 0.05. **(D)** Enrichment of hits in MSigDB Gene Set Expression analysis of genes demonstrates pathways downregulated in colitis compared to non-IBD. Log 2-fold changes > 1.5, FDR p< 0.05. **(E)** Volcano plot reveals enrichment of hits in MSigDB subset analysis (subset “B”) of colitic organoids versus non-IBD organoids. Log 2-fold changes > 1.5, FDR < 0.05. This subset was defined by examining more stringent clustering based on principal component analysis. Increased number of genes is now apparent (>1100 genes), log2 UC/non-IBD, FDR < 0.05 (Benjamini-Hochberg). Red; Colitis, Grey, non-IBD. **(F)** Principal component analysis subset B. Colitic organoids (red) versus non-IBD organoids (blue) for clustered analysis demonstrating a subset of the organoids exhibiting polarized clustering (subset B). **(G)** Gene Set Expression analysis of upregulated genes in subset B compares UC versus non-IBD organoids revealing inflammatory and metabolic pathways. Log2-fold changes > 1.5, FDR < 0.05. **(H)** Gene Set Expression analysis of downregulated genes in subset B comparing UC to non-IBD organoids demonstrates mitochondrial and metabolic pathway enrichment. Log2-fold changes > 1.5, FDR < 0.05.

To identify UC-specific gene expression profiles, total RNA from the non-IBD and UC patient-derived epithelial organoids described earlier was extracted and subjected to next-generation sequencing (RNA-Seq). Differential-expression analysis of the full cohorts of non-IBD and UC organoids, as visualized through volcano plots, highlighted the differing distributions of 260 transcripts detected at an FDR of <0.05 and log2 fold changes of >1.5 (Figure 2A). Of all these 260 genes, 219 (84%) were upregulated in UC organoids compared to control, and 41 (16%) were downregulated. Principal-component (PC) analysis was used to examine the degree of expression profile clustering in each type of organoid. Whereas non-IBD organoids were tightly clustered, UC organoids were widely distributed across the Principal Component (PC1 and PC2) axes (Figure 2B). This high degree of gene expression variation in UC organoids reflected the known morphological heterogeneity of UC and differences in disease presentation.

To examine dysregulation of transcriptional pathways and networks associated with disease processes, the gene expression sets of the UC and non-IBD organoid populations were compared using the Molecular Signatures Database (MSigDB) [19–21]. Gene ontology pathways most significantly associated with transcripts upregulated in UC organoids included cell and anchoring junction, cytoskeleton organization, and tissue development (Figure 2C), and pathways associated with overexpressed transcripts included inflammatory response, development / transmembrane transport of small molecules, focal adhesion, cancer, tissue development, cytoskeleton, cell junction, and anchoring junction (Figure 2D; top-30 upregulated genes in Supplementary Table 5A). In contrast, transcripts downregulated in UC organoids were members of pathways linked to KEGG steroid hormone biosynthesis, metabolism of xenobiotics, Gene Ontology descriptions of protein localization, ion transport, and oxidation/reduction homeostasis pathways (Figure 2D; top-30 downregulated genes in Supplementary Table 5B).

Leveraging another feature of the principal-component analysis, we examined a subset of the entire cohort, which we named “Subset B” (Figure 2E). Here differential-expression analysis identified four UC-organoid isolates that clustered as a clear subpopulation along the PC1 and PC2 axes (Figure 2F; Colitis distal (Ctd) 111, 139, 153, and 155). Repeating the differential-expression analysis of transcripts for these four organoids compared to four highly-clustered non-IBD (NL) organoid isolates (NL 141, 143, 148, 156) revealed a larger and different gene-set enrichment (Figure 2G, 2H). In this analysis, 1,048 differentially expressed transcripts were identified at an FDR of <0.05 and log2 fold changes of >1.5. Transcripts upregulated in Subset B accounted for 864 genes (82%), and downregulated transcripts for 184 genes (18%).

Gene-set enrichment analysis of Subset B using MSigDB [19–21] uncovered further novel disease-associated pathways linked with upregulated genes (Figure 2G). Strong associations were observed with Hallmark TNFα, signaling via NFkB, KEGG toll-like receptor signaling and WNT signaling, reactome metabolism, lipid and lipoprotein metabolic processes and, most prominently, the Gene Ontology indicator for positive regulation of cell differentiation (top-30 upregulated genes in Supplementary Table 6A). Gene sets downregulated in Subset B were involved in Hallmark [20] oxidative phosphorylation, Reactome TCA cycle, respiratory electron transport, and Gene Ontology terms for metabolic processes related to energy metabolism and mitochondrial function (Figure 2H; top-30 downregulated genes Supplementary Table 6B).

These studies thus showed notable differences in the transcriptomes of the UC-derived organoids compared to the transcriptomes from the non-IBD colon organoids. Pronounced differences included altered expression in inflammatory pathways, metabolism, cell adhesion and cancer.

### Profiling of active enhancers identifies genes denoting regeneration and transformation in UC tissue-derived organoids

**Figure 3:**
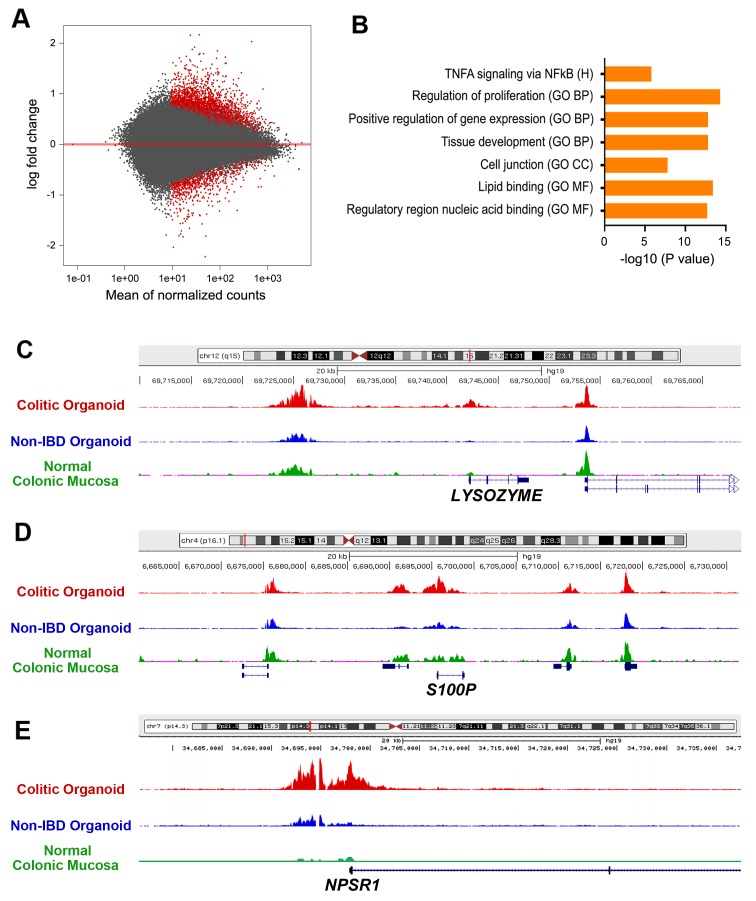
ChIP-seq analysis of H3K27ac enrichment UC versus non-IBD colonic organoids **(A)** Volcano plot representing enrichment by H3K27ac ChIP followed by deep sequencing on a subset of UC versus non-IBD organoids demonstrates differences in expression based on disease, FDR < 0.05. Red: colitis; Grey: non-IBD. **(B)** Molecular signatures database (MSigDB) analyses revealing multiple upregulated gene sets. Log2-fold changes > 1.5, FDR p< 0.05. GO CC: gene ontogeny cellular component; GO BP: gene ontogeny biological process; RCP: reactome curated pathways; H: Hallmark; KCP: KEGG curated pathways; GO MF: Gene ontogeny molecular function. [50] **(C-E)** Normalized H3K27ac ChIP-seq tracks at the *LYZ* (C), *S100P* (D), and *NPSR1* (E) loci, respectively. Red track originates from UC organoids, blue track from non-IBD organoids. Green track is from ENCODE standard for normal colonic mucosa.

Control of gene expression is a complex process that involves coordinated activities of many transcription factors and that is guided by histone modifications in DNA regions serving as *cis*-acting promoters, repressors, enhancers, and insulators [21]. In particular, acetylation H3K27 is positively correlated with increased transcriptional activity at promoter regions at the leading edge of genes, and at enhancer elements located within or nearby genes or sometimes at great distances [15, 16, 22, 23]. To interrogate the epigenetic phenomenon of H3K27 acetylation, a ChIP-Seq analysis was done on five non-IBD (NL) and five UC-epithelial organoid isolates (Ctd). The UC cohort had three organoid isolates belonging to the cluster subclass described earlier, and two UC organoid isolates that were less clustered with this subclass. Ofnote, Ctd150, already recognized as being associated with CAC, was excluded from this analysis. Of the 1,485 region-specific H3K27ac sites identified, 97 were located within 5 kb of the transcription start site (falling largely on promoters and introns), and 894 were 50 to 500 kb away from transcription start sites, and can thus be ascribed to distant enhancers or annotation-deserts. The MA-plot visualizes the differential acetylation in histone H3K27ac across the entire genome comparing UC and non-IBD epithelial organoids at an FDR<0.05 (Figure 3A).

MSigDB analysis of genes within 20 kb of H3K27ac peaks revealed enrichment of genes associated with Hallmark TNFα signaling via NFKB, and the Gene Ontology terms for regulation of proliferation, positive regulation of gene expression, tissue development, cell junction, lipid binding and regulatory region nucleic acid binding (Figure 3B). There was also significant H3K27ac enrichment in DNA regions correlated with cytoskeletal protein binding and transporter activity; top-35 genes associated with significant enrichment of H3K27ac is presented in Supplementary Table 7.

Prominent among genes correlated with enriched H3K27ac activity in UC-epithelial organoids were those that were also upregulated in UC-epithelial organoids in the RNA-Seq studies. For instance, the gene encoding *LYZ*, listed as number 2, was upregulated significantly in the RNA-Seq analysis (Supplementary Table 6). Normalized H3K27ac plots of non-IBD and UC-epithelial organoids illustrated the differing acetylation patterns near the *LYZ* gene between the two populations (listed as number 21 in Supplementary Table 7). Furthermore, ENCODE H3K27ac tracks for normal human colonic mucosa corresponded well with acetylation of enhancer and promoter regions, respectively (Figure 3C). Additional genes that were upregulated in the RNA-Seq experiments and experienced significant H3K27ac enrichment within 20kb of their transcriptional start sites included *S100P*, listed as number 30 for H3K27ac enrichment (Supplementary Table 7) and number 62 within the RNA-seq (Figure 3D, Supplementary Table 6). The protein, S100P, is a calcium-binding protein, which is an established marker of inflammation and correlated with both sporadic colon cancer [24] and ulcerative colitis [25]. *NPSR1*, also called GPR154 or G-protein-coupled receptor for asthma susceptibility, codes for a 7-membrane receptor of unclear function and is likewise associated with susceptibility to inflammatory bowel disease [26] and to neuroendocrine tumors [27]. *NPSR1* is the top 23rd of the H3K27ac enriched gene regions (Supplementary Table 7) and confirmed by the largest log2 fold-change within the entire cohort by RNA-seq (Figure 3E, Supplementary Table 6).

Gene ontogeny via the GREAT [28, 29] analytical tool was then applied to the H3K27ac-enriched ChIP-seq results. Within the cohort tested, the overall ontogeny demonstrated two clusters of enrichment: gastrointestinal neoplasm (p=2.85x10^-11^) and digestive system cancer (p=7.08x10^-11^) (Supplementary Figure 2).

In conclusion, analysis of the chromatin mark H3K27ac marks in UC organoids and non-IBD organoids demonstrates differences in genes associated with bacterial defenses, inflammation, and regeneration. Integrating the transcriptome and the epigenetic H3K27ac enrichment data indicated gene expression signatures consistent with regeneration and gastrointestinal cancer.

### Immunohistochemistry of organoids validates, at the protein level, changes observed at the transcriptional and epigenetic levels in primary organoids and matched tissues from UC, non-IBD and CAC

We then compared protein levels based on findings compiled from RNA-seq and ChIP-seq analyses with individual genes and signaling pathway elements in the organoids and the parental primary tissues. Enhancer enrichment to identify potential drivers of oncologic processes, such as metastases, has been used in murine models of pancreatic cancer [30]. Based on their relative positions noted by enrichment for H3K27ac, confirmed by enrichment in the RNA-seq, and potentially influencing colitis, we chose the triad of *LYZ*, *S100P*, and *NPSR1*. We also compared levels of these three proteins, predicted by the interface of H3K27ac enrichment and RNA-sequencing, in CAC, a devastating sequela of UC, and for which the pathogenesis also remains unclear.

**Figure 4:**
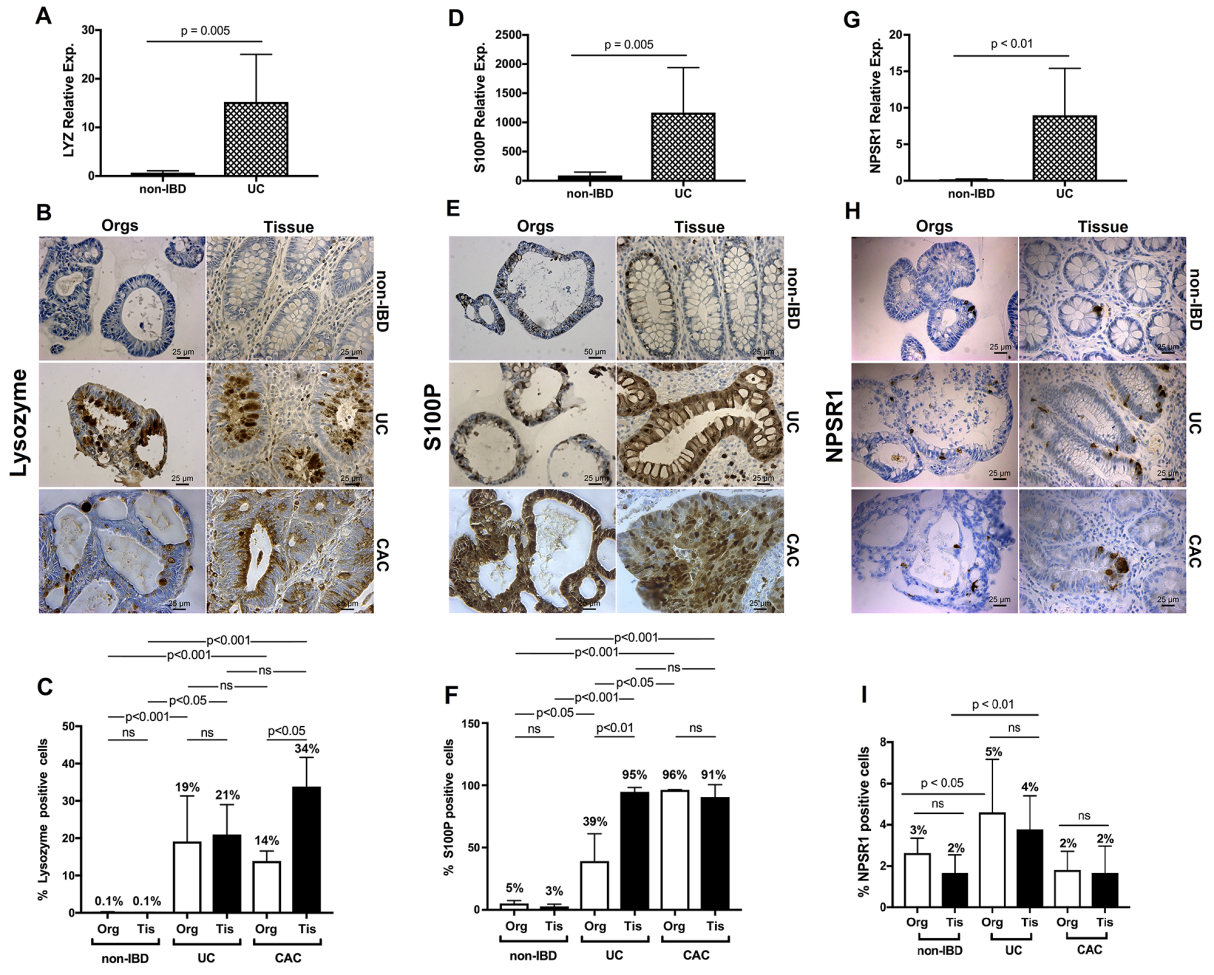
qRT-PCR and IHC validation of enrichment delineated by ChIP-seq and transcriptome analysis for UC and extended to colitis-associated cancer **(A)** *LYZ* quantitative real-time PCR. Quantitative PCR for *LYZ* comparing UC organoids to non-IBD organoids. Relative expression compared to *GAPDH* with relative 2^-ΔΔCT^ expression **(B)** LYZ IHC for non-IBD, colitis-derived, and colitis-associated cancer organoids compared to primary non-IBD, UC and CAC primary tissues. **(C)** Quantification of the percentages of cells positive for LYZ protein. LYZ graphical summaries reflect the differences in enumerated expression. P-value for non-IBD organoids and non-IBD tissues vs. UC and CAC organoids and tissues = 0.0002. **(D-F)** Same data as in panels A – C, for *S100P* reveal increased expression at the transcription and protein levels for both the UC and the CAC organoids. **(G-I)** Same data as for panels A – C for *NPSR1* demonstrates increased expression at the transcriptome and protein levels for both the colitic organoids and tissues but no differences for CAC at the protein level compared to non-IBD tissues. Scale bars as denoted. Colitis, UC; colitis-associated cancer, CAC. N ≥ 4 each. Quantification performed with unpaired two-sided Student’s *t*-test, one-way ANOVA. NS: not significant. At least 4 technical replicates were done except where indicated. Results are represented as mean +/- SEM of ≥ 4 independent experiments.

We first performed qRT-PCR to test whether the RNA-seq and H3K27ac enrichment noted in our studies would be validated (Figure 4A, 4D, and 4G). We compared a subset of the colitic isolates to a subset of the non-IBD isolates. We demonstrated consistency with the cross-sectional analyses for each of the genes in question (*LYZ*, p = 0.005; *S100P*, p < 0.01, and *NPSR1*, p = 0.005, respectively).

LYZ is an antibacterial protein and marker of Paneth cells. These cells, which form the niche of regenerating mucosa, undergo metaplasia and produce large amounts of LYZ during such regenerative processes including recovery from colitic episodes. Consistent with this clinicopathological finding, LYZ expression was enhanced in colitic organoids and tissues, but not in non-IBD organoids and tissues. Further, LYZ is newly identified as bearing enhanced expression in CAC organoids and tissues (Figure 4B–4C).

S100P, a calcium-binding protein engaged as a member of the damage-associated molecular pattern proteins, was also more abundantly expressed in UC and CAC organoids and tissues than in non-IBD organoids and tissues (Figure 4E–4F).

NPSR1, associated with inflammatory bowel disease, showed similar, though less pronounced differences in (i) non-IBD versus (ii) UC and no differences at the level of immunohistochemistry between non-IBD and CAC organoids and tissues (Figure 4H–4I).

In determining whether the organoids might serve as faithful models for the primary tissues, we conducted a second level of analysis. Here significant differences were apparent only between (1) the CAC organoids and tissues expressing LYZ, and (2) the UC organoids and tissues expressing S100P. However, for both proteins, the colitic and CAC organoids along with corresponding tissues expressed significantly more LYZ or S100P, respectively, with p-values < 0.001.

In conclusion, we have validated molecularly defined signatures in both organoids and in parental tissues. Further, we have extended these findings and confirmed the presence of these signatures at the protein level in CAC organoids and tissues, demonstrating increased expression of LYZ and S100P in both colitis and CAC, and increased expression of NPSR1 in colitic organoids and tissues.

## DISCUSSION

We found that epithelial organoids from human UC colon and non-IBD colon exhibit histological phenotypes and molecular signatures that accurately model the states of the primary tissues from which they were derived. Active enhancer profiling, using the H3K27ac mark, coupled with whole-transcriptome analysis of UC and non-IBD organoids, highlighted multiple genes whose expression is altered in UC organoids and that are associated with pathways known to be activated in colitic disease, genes such as *LYZ* and *S100P*. Other genes, including *NPSR1*, were identified as new disease signatures and may be involved in disease pathogenesis. Further, we found that these three proteins are present in CAC. While LYZ has been associated with colitis, and S100P associated with the progression of colitis-associated dysplasia [31], neither LYZ nor NPSR1 had been shown to be associated with CAC. These organoid models were proven here not only to be faithful to the primary disease, but also, helpful in dissecting the etiology of IBD, which involves aberrations in the complex interplay between environment, immune system, and genetics [32]. While recent studies have used cancerous states, such as colonic adenocarcinoma, or precancerous states, such as organoids from familial adenomatous polyposis as tools for understanding oncogenic transformation, for diseased but benign states such as UC, use of primary colonic organoids has been far less common. Therefore, we determined the feasibility of using colon-derived organoids in UC research. Our strategy was to show their fidelity to whole tissues by examining the transcriptome of our two populations of primary organoids. The full complement of organoids examined exhibited transcriptome enrichment profiles consistent with their origins.

Subset analysis showed two distinct clusters: subset B was identified as completely divergent from the non-IBD cluster. Subset B’s transcriptome revealed expression of signaling pathways correlated with the primary colitic state, including upregulation of TNFα, toll-like receptors, and WNT signaling, and downregulation of several metabolic pathways. Several of the downregulated pathways are involved in key aspects of metabolism in mitochondria. Our findings are consistent with findings of others who interrogated whole tissues, examining the whole transcriptome or whole-exome sequencing data, including findings on cellular movement, cytoskeleton, inflammation, WNT signaling, S100P [24, 25] and other proliferative, immune, and inflammatory responses. These studies incorporated whole tissues, while our studies were limited to epithelial-cell enriched organoids. We also corroborated other studies that included evaluation of formalin-fixed, paraffin-embedded, micro-dissected CAC sections by demonstrating increased expression of Rho and Rac pathways [8].

We initiated our validation studies with examination of LYZ, whose expression correlates with Paneth cells, which in addition to bearing antibacterial activity, form the niche initiating tissue regeneration at the crypt base [33, 34]. We first correlated expression of LYZ in UC and non-IBD organoids. As expected, non-IBD epithelial organoids were virtually devoid of staining for the Paneth cell marker LYZ, but, immunohistochemical staining of UC-derived epithelial organoids for LYZ demonstrated metaplastic expression of this cell type. Paneth cell metaplasia of the colon is pathognomonic for UC, as these cells are virtually non-existent in a normal colon [33, 34]. Therefore, it is not surprising that colitic organoids phenocopy this state and express LYZ.

S100P is a member of a family of calcium-binding proteins that signal via the receptor for advanced glycation end products [24, 25, 35–37]. Others have noted increased expression of S100P in colitis, adenomas and sporadic colon cancer. In some cases, S100P expression correlated with progression of colitis into CAC [36]. Our findings confirm these observations in the context of non-IBD colitis, and extend those findings to CAC.

*NPSR1* or *GPR154* was identified by examination of the H3K27ac-enriched regions and RNA levels. While there have been reports of isoforms of this gene associated with inflammatory bowel disease, and especially with ulcerative colitis, further mechanistic studies to uncover functional linkages either for colitis or for CAC have been lacking. However, the association and mechanisms for how *NPSR1* signals in other immune-mediated phenotypes such as asthma [38], psoriasis [39], and allergies [40] as well as downstream signaling in inflammatory cascades that imply similar propensities may occur in UC [27]. Furthermore, the association with neuroendocrine tumors and downstream inflammatory signaling cascades including the mitogen-activated protein kinase pathways indicate potential inflammatory drivers towards malignancy, not unlike that which may occur with CAC [27]. Validation of the expression of *NPSR1* in colitic organoids was supported by the observation that H3K27ac marked this gene, and by immunohistochemistry data in both UC organoids and corresponding tissues.

In conclusion, our data have demonstrated the feasibility and usefulness of establishing primary organoids from normal, UC and CAC human samples. They have substantiated that LYZ has a role in UC and have now newly identified a role for LYZ in CAC. Previous studies indicating a role of S100P in colitis and CAC were also validated [24, 25, 35–37]. We identified NPSR1 as a potential effector in the pathogenesis of UC. Combining these sequencing technologies with organoid models may be used to facilitate mechanistic gaps in diseases where the pathogenesis is challenging to discern [30]. Indeed, organoids are now being used to establish drug sensitivities, resistance, and mechanisms for sporadic colorectal cancer [10, 41]. Such studies are expected to greatly facilitate the dissection of pathogenic mechanisms in UC and CAC, with the ultimate goal of allowing the use of precision medicine for these devastating diseases [42].

## MATERIALS AND METHODS

### Patient population; tissue collection

Tissue specimens were obtained from distal colons of 10 UC patients who underwent colectomy for unremitting disease. Patient demographics and clinical presentations are summarized in Supplementary Table 1. None had strictures or sclerosing cholangitis, known risk factors for CAC. As UC patients often have pan-colitis, manifested as inflammation along the entire tract of the large bowel, matched “normal” tissue specimens from these 10 UC patients were not used to cultivate control (non-diseased) colonic organoids. Instead, healthy normal colonic tissue was obtained from 10 patients who had undergone colonic resection for reasons other than IBD (i.e., colon cancer). Pathology of retrieved tissues was confirmed by Cleveland Clinic’s pathology department and approved via IRB 13-1159.

### Cultivation of patient-derived colonic epithelial organoids

Intestinal epithelial organoids were cultivated in Matrigel^®^ beads according to VanDussen [12] using 50% Advanced DMEM and 50% conditioned medium conditioned by Wnt3a, R-spondin 3 and Noggin (L-WRN) L-cells, prepared according to Miyoshi and Stappenbeck [43].

Epithelial phenotypes of cultured organoids were established by immunocytochemical staining for cytokeratin 19 (CK19; epithelial-cell marker) and the absence of staining for vimentin (VIM; mesenchymal marker) (Supplementary Figure 1A-1D).

Short tandem repeat (STR) [44] analysis of genomic DNA extracted from each patient-derived organoid line validated the unique patient origin (Supplementary Table 2).

### Microscopy and immunohistochemistry/immunofluorescence

Intestinal epithelial organoids were prepared for microscopy by release from Matrigel^®^ beads through gentle washing in PBS, immediate fixation with 4% paraformaldehyde, and embedding in 1.5% agarose. Standard conditions – with tris/borate/EDTA buffer (Discovery CC1, 950-500; Ventana) on a Discovery ULTRA automated stainer from Ventana Medical System Inc (Tucson, AZ) – were used. Chamber slides with acetone-fixed epithelioids were evaluated for CK19 and VIM antibodies listed in Supplementary Table 3. Images were captured on a Leica microsystems microscope (version 4.3.0). Enumeration of cells expressing these proteins was done manually (>500 nuclei/section/stain).

Cytokeratin 19 and Vimentin IF stains for epithelioids and potential stroma were completed in suspension. Briefly, organoids were fixed for 1-3 h in 4% paraformaldehyde, treated with blocking serum (5% serum in 1X PBS plus 0.5% Triton-X100) for 1 h, then exposed to primary antibodies, washed in PBS-0.5% Triton-X100, and exposed to a secondary antibody. Suspended organoids in a drop of VECTASHIELD mounting medium with DAPI for staining nuclei, were loaded on the coverWell imaging chamber (Cat no. C18160, ThermoFisher Scientific) for confocal microscopy. Images were captured on a Leica microsystems confocal microscope (DM16000) using 2.7.3.9723 version of the software, magnification x40, oil immersion (Supplementary Figure 1A-1D).

### RNA Isolation

Intestinal epithelial organoids exhibiting full mini-gut architecture (∼3 wks in continuous culture) were harvested by washing with cold PBS and low-speed centrifugation (200x*g*, 5 min). Total RNA was extracted using miRNeasy kits (Qiagen). RNA purity/integrity was assessed using a Bioanalyzer (Agilent).

### RNA sequencing (RNA-Seq)

Total RNA was processed for next-generation sequencing using TruSeq Total RNA kits (Illumina). Total RNA of each sample was depleted of ribosomal RNA using biotinylated oligomers, combined with Ribo-Zero rRNA removal beads and fragmentation of RNA using divalent cations at elevated temperatures. Cleaved RNA fragments were copied into double-stranded cDNA using (sequentially) reverse transcriptase, random primers, DNA polymerase, and RNase H. Following ligation of the sequencing adapter, the cDNA products were purified. Paired end 100-bp sequencing reads generated from the Illumina HiSeq-2500 platform were assessed for quality and trimmed (https://github.com/FelixKrueger/TrimGalore). Alignment was made to the human genome (GRCh38 assembly) using the Tuxedo package [45] followed by analysis for differential expression using a significance cut-off of a false discovery rate (FDR) <0.05 and a log2 fold change of >1.5 or < -1.5.

### Chromatin immunoprecipitation

Chromatin immunoprecipitation and preparation of DNA fragments enriched for H3K27 acetylation was done as reported [15, 16, 46]. The specificity of immunoprecipitation was validated by qPCR amplification of sample DNA using primers for genomic regions (Supplementary Table 4) either enriched in or devoid of H3K27ac marks based on ENCODE data [47] (Supplementary Figure 3). Bioanalyzer analysis of fragments post enrichment and prior to sequencing revealed appropriately sized fragments (Supplementary Figure 4).

### Library preparation and deep sequencing

Input DNA and H3K27ac-antibody-enriched DNA were quantified by PicoGreen (Invitrogen) and libraries were prepared using ThruPLEX^®^ Illumina next-generation sequencing (NGS) library preparation kits (Rubicon) [15].

ChIP-seq reads were confirmed for quality using FastQC, trimmed (Trim Galore), and aligned to the GRCh19 human genome assembly using Bowtie2 [47]. Aligned chromatin immunoprecipitated reads were then normalized to whole-cell extract input DNA reads using BamTools (Galaxy). Peak calls were performed using MACS2 software. Regional differences in H3K27ac activity were analyzed for statistical significance using the DESeq2 package and p<0.05 [15, 48, 49].

### Real-time PCR

Organoids were extensively washed and Total RNA was extracted using the miRNeasy kit method (Qiagen #217004). Purity and integrity of the extracted RNA was assessed using the Agilent Bioanalyzer, and only samples with RIN values greater than 8.0 were used for gene expression analysis. Total RNA underwent RNase–free DNase I treatment using either on-column treatment (Qiagen; #79254) or Amplification grade DNase I (Invitrogen Life Science Technologies, #18068-015) and was then processed into cDNA using iScript^®^ cDNA Synthesis Kit according to the manufacturer’s specifications (Bio-Rad; #1708891). The resulting amplified cDNA underwent an additional round of amplification using the Ovation Pico WTA System V2 (NuGEN; #3302-60-NUG). TaqMan expression assays from Applied Biosystems were used to detect *LYZ*(Hs00426232_m1), *S100P* (Hs00195584_m1), *NPSR1* (Hs01036497_m1), and the house-keeping *GAPDH* (Hs02786624_m1) gene, on an Applied Biosystems Model 7500 or 7900 Real Time PCR instrument equipped with SDS Version 2.3 and 2.4 software, respectively. Data were normalized using GAPDH and underwent comparative CT analysis using the 2ΔΔCT method.

### Statistics

Two-sample *t*-tests were done to compare the % of positive cells in normal and colitis samples for *MUCIN* 2 (MUC2), *LYZ*, *S100P*, and *NPSR1* in organoid and tissue specimens. Paired *t*-tests were used to investigate the validity of the organoid model by comparing the % of positively stained cells in tissue and organoid samples for the same gene and disease status. P <0.05 was considered significant. The Graph-Pad Prism 7 statistical package was used to graph the data and determine significance.

### GEO Database submission

RNA-seq and ChIP-seq data can be found in the GEO database GSE102746 and GSE 103476, respectively.

## SUPPLEMENTARY MATERIALS FIGURES AND TABLES



## Abbreviations

CAC: Colitis-associated cancer
ChIP-Seq: Chromatin Immunoprecipitation Sequencing
Ctd: Colitis distal
CK19: Cytokeratin19
FDR: False Discovery Rate
GO BP: Gene ontogeny biological process
GO CC: Gene ontogeny cellular component
GO MF: Gene ontogeny molecular function
GSEA: Gene sequence enrichment analysis
H: Hallmark database
IBD: inflammatory bowel disease
KCP: KEGG curated pathways
KEGG: Kyoto encyclopedia of genes and genomes
L-WRN: L-cell line to secrete Wnt3a, R-spondin 3 and Noggin
LYZ: Lysozyme
MSigDB: Molecular Signaling Database
MUC2: Mucin 2
NGS: Next Generation Sequencing
NPSR1: Neuropeptide S Receptor 1
PBS: phosphate-buffered saline
RCP: Reactome curated pathways
RNA-Seq: RNA sequencing (RNA-Seq)
S100P: Calcium-Binding Protein P
STR: short tandem repeat
TNFα: Tumor Necrosis Factor Alpha
UC: ulcerative colitis
VIM: Vimentin

## References

[1] Danese S, Fiocchi C (2011). Ulcerative colitis. N Engl J Med.

[2] Dorofeyev AE, Vasilenko IV, Rassokhina OA, Kondratiuk RB (2013). Mucosal barrier in ulcerative colitis and Crohn’s disease. Gastroenterol Res Pract.

[3] Dotti I, Mora-Buch R, Ferrer-Picon E, Planell N, Jung P, Masamunt MC, Leal RF, Martin de Carpi J, Llach J, Ordas I, Batlle E, Panes J, Salas A (2017). Alterations in the epithelial stem cell compartment could contribute to permanent changes in the mucosa of patients with ulcerative colitis. Gut.

[4] McCole DF (2014). IBD candidate genes and intestinal barrier regulation. Inflamm Bowel Dis.

[5] Anderson CA, Boucher G, Lees CW, Franke A, D’Amato M, Taylor KD, Lee JC, Goyette P, Imielinski M, Latiano A, Lagace C, Scott R, Amininejad L (2011). Meta-analysis identifies 29 additional ulcerative colitis risk loci, increasing the number of confirmed associations to 47. Nat Genet.

[6] Cho JH, Brant SR (2011). Recent insights into the genetics of inflammatory bowel disease. Gastroenterology.

[7] Cardinale CJ, Wei Z, Li J, Zhu J, Gu M, Baldassano RN, Grant SF, Hakonarson H (2014). Transcriptome profiling of human ulcerative colitis mucosa reveals altered expression of pathways enriched in genetic susceptibility loci. PLoS One.

[8] Planell N, Lozano JJ, Mora-Buch R, Masamunt MC, Jimeno M, Ordas I, Esteller M, Ricart E, Pique JM, Panes J, Salas A (2013). Transcriptional analysis of the intestinal mucosa of patients with ulcerative colitis in remission reveals lasting epithelial cell alterations. Gut.

[9] Khor B, Gardet A, Xavier RJ (2011). Genetics and pathogenesis of inflammatory bowel disease. Nature.

[10] Li X, Ootani A, Kuo C (2016). An Air-Liquid Interface Culture System for 3D Organoid Culture of Diverse Primary Gastrointestinal Tissues. Methods Mol Biol.

[11] Sato T, Stange DE, Ferrante M, Vries RG, Van Es JH, Van den Brink S, Van Houdt WJ, Pronk A, Van Gorp J, Siersema PD, Clevers H (2011). Long-term expansion of epithelial organoids from human colon, adenoma, adenocarcinoma, and Barrett’s epithelium. Gastroenterology.

[12] VanDussen KL, Marinshaw JM, Shaikh N, Miyoshi H, Moon C, Tarr PI, Ciorba MA, Stappenbeck TS (2015). Development of an enhanced human gastrointestinal epithelial culture system to facilitate patient-based assays. Gut.

[13] McGovern DP, Gardet A, Torkvist L, Goyette P, Essers J, Taylor KD, Neale BM, Ong RT, Lagace C, Li C, Green T, Stevens CR, Beauchamp C (2010). Genome-wide association identifies multiple ulcerative colitis susceptibility loci. Nat Genet.

[14] Guenther CA, Tasic B, Luo L, Bedell MA, Kingsley DM (2014). A molecular basis for classic blond hair color in Europeans. Nat Genet.

[15] Mack SC, Witt H, Piro RM, Gu L, Zuyderduyn S, Stutz AM, Wang X, Gallo M, Garzia L, Zayne K, Zhang X, Ramaswamy V, Jager N (2014). Epigenomic alterations define lethal CIMP-positive ependymomas of infancy. Nature.

[16] Cohen AJ, Saiakhova A, Corradin O, Luppino JM, Lovrenert K, Bartels CF, Morrow JJ, Mack SC, Dhillon G, Beard L, Myeroff L, Kalady MF, Willis J (2017). Hotspots of aberrant enhancer activity punctuate the colorectal cancer epigenome. Nat Commun.

[17] Gersemann M, Becker S, Kubler I, Koslowski M, Wang G, Herrlinger KR, Griger J, Fritz P, Fellermann K, Schwab M, Wehkamp J, Stange EF (2009). Differences in goblet cell differentiation between Crohn’s disease and ulcerative colitis. Differentiation.

[18] Massironi S, Zilli A, Cavalcoli F, Conte D, Peracchi M (2016). Chromogranin A and other enteroendocrine markers in inflammatory bowel disease. Neuropeptides.

[19] Liberzon A (2014). A description of the Molecular Signatures Database (MSigDB) Web site. Methods Mol Biol.

[20] Liberzon A, Birger C, Thorvaldsdottir H, Ghandi M, Mesirov JP, Tamayo P (2015). The Molecular Signatures Database (MSigDB) hallmark gene set collection. Cell Syst.

[21] Liberzon A, Subramanian A, Pinchback R, Thorvaldsdottir H, Tamayo P, Mesirov JP (2011). Molecular signatures database (MSigDB) 3.0. Bioinformatics.

[22] Kimura H (2013). Histone modifications for human epigenome analysis. J Hum Genet.

[23] Akhtar-Zaidi B, Cowper-Sal-lari R, Corradin O, Saiakhova A, Bartels CF, Balasubramanian D, Myeroff L, Lutterbaugh J, Jarrar A, Kalady MF, Willis J, Moore JH, Tesar PJ (2012). Epigenomic enhancer profiling defines a signature of colon cancer. Science.

[24] Fuentes MK, Nigavekar SS, Arumugam T, Logsdon CD, Schmidt AM, Park JC, Huang EH (2007). RAGE activation by S100P in colon cancer stimulates growth, migration, and cell signaling pathways. Dis Colon Rectum.

[25] Lawrance IC, Fiocchi C, Chakravarti S (2001). Ulcerative colitis and Crohn’s disease: distinctive gene expression profiles and novel susceptibility candidate genes. Hum Mol Genet.

[26] D’Amato M, Bruce S, Bresso F, Zucchelli M, Ezer S, Pulkkinen V, Lindgren C, Astegiano M, Rizzetto M, Gionchetti P, Riegler G, Sostegni R, Daperno M (2007). Neuropeptide s receptor 1 gene polymorphism is associated with susceptibility to inflammatory bowel disease. Gastroenterology.

[27] Pulkkinen V, Ezer S, Sundman L, Hagstrom J, Remes S, Soderhall C, Greco D, Haglund C, Kere J, Arola J (2014). Neuropeptide S receptor 1 (NPSR1) activates cancer-related pathways and is widely expressed in neuroendocrine tumors. Virchows Arch.

[28] McLean CY, Bristor D, Hiller M, Clarke SL, Schaar BT, Lowe CB, Wenger AM, Bejerano G (2010). GREAT improves functional interpretation of cis-regulatory regions. Nat Biotechnol.

[29] Osborne JD, Flatow J, Holko M, Lin SM, Kibbe WA, Zhu LJ, Danila MI, Feng G, Chisholm RL (2009). Annotating the human genome with Disease Ontology. BMC Genomics.

[30] Roe JS, Hwang CI, Somerville TDD, Milazzo JP, Lee EJ, Da Silva B, Maiorino L, Tiriac H, Young CM, Miyabayashi K, Filippini D, Creighton B, Burkhart RA (2017). Enhancer Reprogramming Promotes Pancreatic Cancer Metastasis. Cell.

[31] Brentnall TA, Pan S, Bronner MP, Crispin DA, Mirzaei H, Cooke K, Tamura Y, Nikolskaya T, Jebailey L, Goodlett DR, McIntosh M, Aebersold R, Rabinovitch PS, Chen R (2009). Proteins that underlie neoplastic progression of ulcerative colitis. Proteom ICS Clin Appl.

[32] Sartor RB (2006). Mechanisms of disease: pathogenesis of Crohn’s disease and ulcerative colitis. Nat Clin Pract Gastroenterol Hepatol.

[33] Simmonds N, Furman M, Karanika E, Phillips A, Bates AW (2014). Paneth cell metaplasia in newly diagnosed inflammatory bowel disease in children. BMC Gastroenterol.

[34] Tanaka M, Saito H, Kusumi T, Fukuda S, Shimoyama T, Sasaki Y, Suto K, Munakata A, Kudo H (2001). Spatial distribution and histogenesis of colorectal Paneth cell metaplasia in idiopathic inflammatory bowel disease. J Gastroenterol Hepatol.

[35] Logsdon CD, Fuentes MK, Huang EH, Arumugam T (2007). RAGE and RAGE ligands in cancer. Curr Mol Med.

[36] Prica F, Radon T, Cheng Y, Crnogorac-Jurcevic T (2016). The life and works of S100P - from conception to cancer. Am J Cancer Res.

[37] Turovskaya O, Foell D, Sinha P, Vogl T, Newlin R, Nayak J, Nguyen M, Olsson A, Nawroth PP, Bierhaus A, Varki N, Kronenberg M, Freeze HH (2008). RAGE, carboxylated glycans and S100A8/A9 play essential roles in colitis-associated carcinogenesis. Carcinogenesis.

[38] Couzin J (2004). Genetics. Two new asthma genes uncovered. Science.

[39] Kere J (2005). Mapping and identifying genes for asthma and psoriasis. Philos Trans R Soc Lond B Biol Sci.

[40] Orsmark-Pietras C, Melen E, Vendelin J, Bruce S, Laitinen A, Laitinen LA, Lauener R, Riedler J, von Mutius E, Doekes G, Wickman M, van Hage M, Pershagen G (2008). Biological and genetic interaction between tenascin C and neuropeptide S receptor 1 in allergic diseases. Hum Mol Genet.

[41] Sato T, Clevers H (2013). Growing self-organizing mini-guts from a single intestinal stem cell: mechanism and applications. Science.

[42] Pauli C, Hopkins BD, Prandi D, Shaw R, Fedrizzi T, Sboner A, Sailer V, Augello M, Puca L, Rosati R, McNary TJ, Churakova Y, Cheung C (2017). Personalized *in vitro* and *in vivo* cancer models to guide precision medicine. Cancer Discov.

[43] Miyoshi H, Stappenbeck TS (2013). *in vitro* expansion and genetic modification of gastrointestinal stem cells in spheroid culture. Nat Protoc.

[44] Chen S, Fisher RC, Signs S, Molina LA, Shenoy AK, Lopez MC, Baker HV, Koomen JM, Chen Y, Gittleman H, Barnholtz-Sloan J, Berg A, Appelman HD, Huang EH (2016). Inhibition of PI3K/Akt/mTOR signaling in PI3KR2-overexpressing colon cancer stem cells reduces tumor growth due to apoptosis. Oncotarget.

[45] Sangwung P, Zhou G, Nayak L, Chan ER, Kumar S, Kang DW, Zhang R, Liao X, Lu Y, Sugi K, Fujioka H, Shi H, Lapping SD (2017). KLF2 and KLF4 control endothelial identity and vascular integrity. JCI Insight.

[46] Miller TE, Liau BB, Wallace LC, Morton AR, Xie Q, Dixit D, Factor DC, Kim LJY, Morrow JJ, Wu Q, Mack SC, Hubert CG, Gillespie SM (2017). Transcription elongation factors represent *in vivo* cancer dependencies in glioblastoma. Nature.

[47] Sloan CA, Chan ET, Davidson JM, Malladi VS, Strattan JS, Hitz BC, Gabdank I, Narayanan AK, Ho M, Lee BT, Rowe LD, Dreszer TR, Roe G (2016). ENCODE data at the ENCODE portal. Nucleic Acids Res.

[48] Burden CJ, Qureshi SE, Wilson SR (2014). Error estimates for the analysis of differential expression from RNA-seq count data. PeerJ.

[49] Ghosh S, Chan CK (2016). Analysis of RNA-Seq data using tophat and cufflinks. Methods Mol Biol.

[50] Gough J, Karplus K, Hughey R, Chothia C (2001). Assignment of homology to genome sequences using a library of hidden Markov models that represent all proteins of known structure. J Mol Biol.

