# Supplementary material for: Cancer-predicting transcriptomic and epigenetic signatures revealed for ulcerative colitis in patient-derived epithelial organoids
Source: Oncotarget. 2018 Jun 19;9(47):28717–30. doi: 10.18632/oncotarget.25617 (PMC6033374; doi:10.18632/oncotarget.25617)
Supplement: Supplementary file 1 [file oncotarget-09-28717-s001.pdf]

Cancer-predicting transcriptomic and epigenetic signatures revealed for ulcerative colitis in patient-derived epithelial organoids

SUPPLEMENTARY MATERIALS

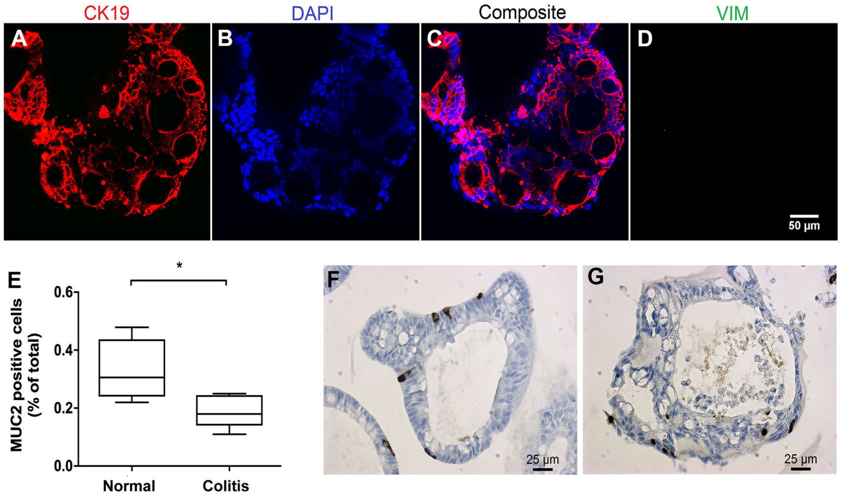

Supplementary Figure 1: Colitis-derived versus non-IBD organoids. **(A-D)** Confocal image confirms the epithelial nature of the organoids and absence of mesenchyme. Immunofluorescence for epithelial marker, CK19 (red, panels A, C) is present, while the mesenchymal marker, VIM (panel D), is absent. DAPI, blue. Scale bar; 50 µm. N > 20. **(E)** Quantification of MUC2 protein level in colitic versus non-IBD (normal) colon organoid. *; $p < 0.05$. **(F, G)** Immunohistochemistry for chromogranin A. Non-IBD colon organoid versus colitis derived organoid. Staining is equivalent (data not shown), N > 9.

Disease Ontology (20+ terms) Global controls

Table controls: Export Shown top rows in this table: 20 Set Term annotation count: Min: 1 Max: Inf Set Visualize this table: [select one]

Term Name	Binom Rank	Binom Raw P-Value	Binom FDR Q-Val	Binom Fold Enrichment	Binom Observed Region Hits	Binom Region Set Coverage	Hyper Rank	Hyper FDR Q-Val	Hyper Fold Enrichment	Hyper Observed Gene Hits	Hyper Total Genes	Hyper Gene Set Coverage
gastrointestinal neoplasm	2	2.8487e-11	3.1835e-8	2.2385	81	9.78%	16	3.6398e-4	2.0809	45	384	4.43%
digestive system cancer	4	7.0816e-11	3.9568e-8	2.1958	81	9.78%	17	4.5210e-4	2.0594	45	388	4.43%

Supplementary Figure 2: Gene Ontogeny GREAT Chip-seq analysis. This figure is extracted from the analysis revealing overall gene expression changes in UC versus non-IBD patient-derived organoids towards a transformed gastrointestinal pathway.

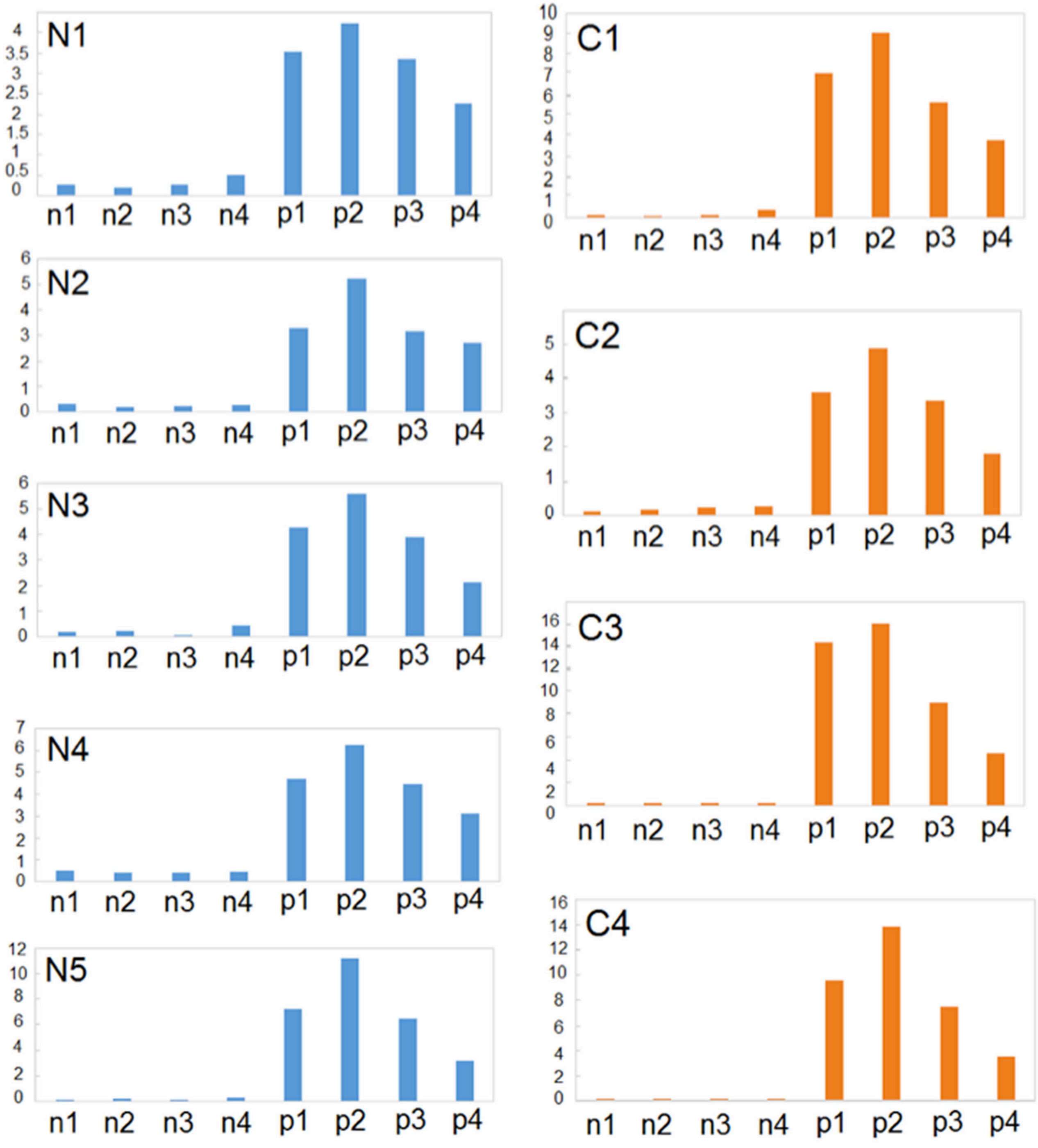

Supplementary Figure 3: H3K27ac enrichment confirmation. After enrichment via ChIP for H3K27ac, qRT-PCR demonstrates enrichment for primers within the H3K27ac region (primers p1-p4) with absence of non-specific transcripts (primers n1 – n4). Primer pairs are listed in Supplementary Table 4.

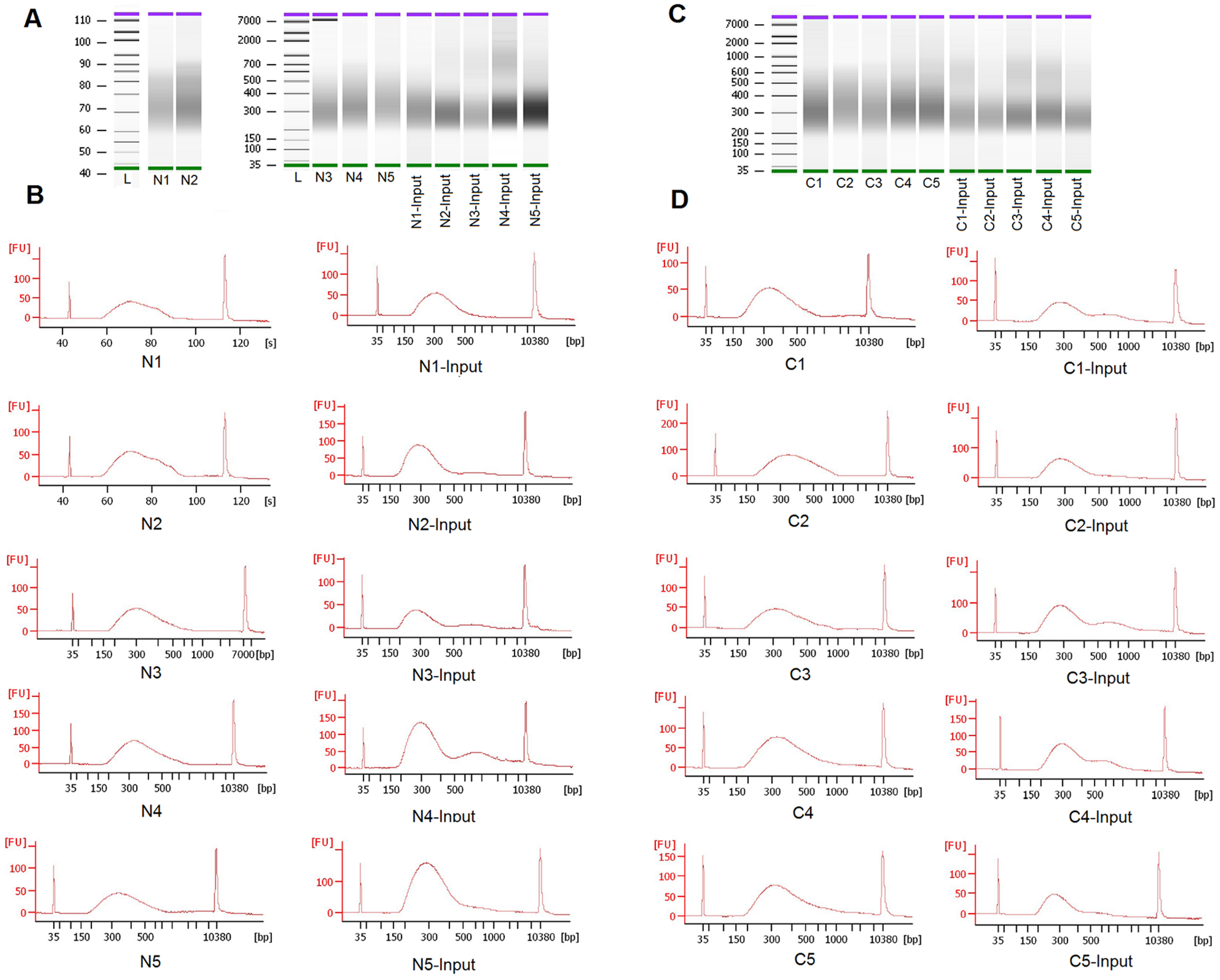

Supplementary Figure 4: Bioanalyzer results for H3K27ac enriched fragments after library preparation and prior to ChIP-seq analysis. **(A)** Bioanalyzer gel demonstrates fragments from non-IBD organoids after chromatin immunoprecipitation (N1–N5) and prior to immunoprecipitation (N1-N5-input). **(B)** Non-IBD average base pair (bp) size demonstrates the majority of input and post-ChIP output to the preferred range size of 200 – 500 bps. **(C)** Bioanalyzer gel demonstrates fragments from UC organoids after chromatin immunoprecipitation (C1– C5) and prior to immunoprecipitation (C1-C5-input). **(D)** UC average base pair (bp) size demonstrates the majority of input and post-ChIP output to the preferred range size of 200 – 500 bps.

Supplementary Table 1: Patient demographics

Patient ID	Indication for Surgery	Pathology (overall)	M/F	Race	Disease Duration (Years)	Age at resection
NI 135	Colon cancer	Normal	F	White	NA	55
NI 137	Colon cancer	Normal	M	White	NA	65
NI 141	Colon cancer	Normal	F	White	NA	73
NI 143	Colon cancer	Normal	M	White	NA	74
NI 144	Colon cancer	Normal	F	White	NA	66
NI 145	Colon cancer	Normal	F	White	NA	89
NI 147	Colon cancer	Normal	M	White	NA	60
NI 148	Colon cancer	Normal	M	White	NA	76
NI 149	Colon cancer	Normal	M	White	NA	65
NI 156	Colon cancer	Normal	M	White	NA	62
CTd 111	Medically refractory colitis	Low-grade dysplasia	M	White	5	60
CTd 139	Medically refractory colitis	Colitis	F	White	3	44
CTd 140	Medically refractory colitis	Colitis with benign strictures	M	White	36	58
CTd 142	Medically refractory colitis	Chronic active colitis	M	White	3	18
CTd 146	Medically refractory colitis	Colitis	F	White	19	58
CTd 150	Cancer/Colitis	T3N1a	F	White	13	44
CTd 151	Medically refractory colitis	Chronic active colitis	F	White	3	40
CTd 152	Medically refractory colitis	Colitis with fibrosis and treatment effect	M	White	6	25
CTd 153	Medically refractory colitis	Chronic active colitis	M	White	6	24
CTd 155	Medically refractory colitis	Chronic active colitis	M	White	5	50

Patient indication for surgery, pathology, gender, ethnicity, disease duration, age at resection.

Supplementary Table 2: Short-tandem repeat: Analysis of genomic DNA extracted from epithelioids

Patient	D13S317	D16S539	CSF1PO	TH01	vWA	D21S11	D7S820	D5S818	TPOX	Amelogenin
NI 135 EP	10,13	12	9,12	6,7	17	32.2	10,12	9,13	8,10	X
NI 137 EP	11	12,14	11,13	9.3	16,17	30,30.2	8,10	11,12	8,9	X,Y
NI 141 EP	11,12	9,12	11	9,9.3	17,19	28,30	9,13	10,13	8,11	X, Y
NI 143 EP	11,12	11	10,12	6,9	17	28,29	10	11,14	8,9	X,Y
NI 144 EP	11	11,13	11,12	9.3	16,19	29,31.2	10	11	6,11	X
NI 145 EP	11	8,13	10,13	6,8	17,18	27,29	10,12	12,13	8	X,Y
NI 147 EP	11,13	11,12	10,13	6,9.3	14,17	30,30.2	8,10	12,13	9,11	X,Y
NI 148 EP	11	11,14	11,13	5,7	14,16	30,33.2	9,10	10,11	10,11	X,Y
NI 149 EP	8,12	11,12	9,12	8,9.3	14,15	32.2,33.2	9,10	12	8,10	X,Y
NI 156 EP	12,13	9,11	10,12	8,9	18,20	28,32.2	8,10	13	11	X,Y
CTd 111 EP	9,12	13	11,12	6,9	14,18	29,30.2	9,12	11,12	10,11	X,Y
CTd 139 EP	12	13	11,12	9	17,18	30,31.2	10,11	12,13	8,9	X
CTd 140 EP	12	11,14	12,13	7,9	15,16	32.2	10,11	9,12	8,11	X,Y
CTd 142 EP	11,	11	8,11	7,9.3	16,17	30,33	9,11	13	9,11	X
CTd 146 EP	8,9	9	10,12	7,9	15,17	28,30	8,10	12,13	8,11	X
CTd 150 EP	11,13	12,13	10,12	9.3	14,17	29	8,9	11,12	8,11	X
CTd 151 EP	11,12	12	9,12	6,7	18	27,30	10,13	10,13	8,9	X
CTd 152 EP	11,12	9,11	10	6.9.3	17	28,30	8,12	11	8,11	X,Y
CTd 153 EP	8,9	11,12	9,12	6,9.3	14,17	31,33.2	9,10	11	9,11	X,Y
CTd 155 EP	8	12,13	11,12	7,9.3	17	30,31.2	8,10	12,13	8,11	X,Y

Supplementary Table 3: Immunohistochemistry antibodies, vendors, concentrations

Antigen	Vendor	Catalog Number	Dilution
S100P	Abcam	ab133654	1/200
PHLDA1	Abcam	ab133654	1/50
ALDH1A1	Abcam	ab52492	1/60
Ki67	Novus	NB110-89717	1/1000
MUC2	Abcam	ab7848	1/400
Lysozyme	Dako	A0099	1/100
Cytokeratin 19	Abcam	ab8187	1/400
Vimentin	R&D systems	MAB2105	1/1000

Supplementary Table 4: Gene enrichment confirmatory primers for H3K27ac*

Name	Forward primer	Reverse Primer
THAP9 - N1	CCAGCCAGGTTTTCATCATT	TGCAGCAACATGGATAGAGC
CHD1 - N1	GGGTGAAGCTGACTCTGAGG	CAGCCTGGGCAATATAGGAA
CHD1 - N2	CCCCTCAAGACCACACCTAA	GTGTGCATCCTCCCAAAACT
CHD1 - P1	GGTATGGGCGCTAATGTCAC	GAGTCCCATCGCAGTCTCTC
SOX4 - P1	AAGCTTCAGCAACCAGCATT	CCCTCTCTCTCGCTCTCTCA
RFX3 - P1	CTCTCATCCCCCGTTACTCA	GGTGTTTGAGATGGCAGGTT
KLF6 - N1	CAGAGGGGAAGCATGCTAAG	ACCGGGATTCGAAAATTACC
KLF6 - P2	AGAGGCAGCAGCGATGTATT	GTGCTCATCAGCGTAATGGA

*In addition to comparison to input DNA, these primers were used to validate specificity of enrichment.

Supplementary Table 5: Full cohort RNA-seq upregulated and downregulated genes (Represented top 30 upregulated and downregulated genes, comparing colitis to non-IBD colon epithelioids)

A. Top 30 upregulated genes full cohort		B. Top 30 downregulated genes full cohort	
**Symbol**	**Log2 (FC)**	**Symbol**	**Log2 (FC)**
SLC28A2	3.02187	SYNGAP1	-2.4198
APOL1	2.47946	UGT2A3	-1.75048
PPDPF	2.47631	RETNLB	-1.5931
AQP3	2.46268	PLA2G4C	-1.43669
HOXB13	2.44272	ENPP3	-1.41561
ARHGDIA	2.25761	RPL9	-1.35255
FBRSL1	2.21881	RYR3	-1.13516
PLA2G2A	2.05797	C1orf132	-1.10852
HNRNPH3	2.04738	C8orf4	-1.0697
HAGHL	1.98728	STIM1	-1.04205
PODXL	1.97956	TM4SF1	-0.98237
MYD88	1.96608	AKR1C3	-0.95896
NRBP2	1.95469	AKR1C2	-0.9528
WDR72	1.94592	ERO1A	-0.93548
ZYX	1.93319	GABRA2	-0.92556
RPLP1	1.91376	ZG16	-0.89103
TIMP4	1.90854	ARRDC3	-0.84477
UBALD1	1.90315	SLC11A2	-0.84332
BCL9L	1.90288	GOLT1A	-0.84284
PHLDA2	1.89078	TLR4	-0.7723
ATP2A3	1.87201	MT-ND6	-0.75765
HSPB1	1.86269	FTL	-0.75299
KRT7	1.85622	ANXA2	-0.73331
SLC7A7	1.84861	P4HA1	-0.73034
CLDN3	1.83269	SAT1	-0.72137
MAST3	1.79142	VWA5A	-0.70748
ATP6V0C	1.78948	ALDH1A1	-0.70505
NFIC	1.7841	PTTG1	-0.69391
UBALD2	1.77664	NOP10	-0.68687
TTLL6	1.76493	RPL34	-0.67344

Supplementary Table 6: Top upregulated and downregulated genes subset B

A. Top upregulated genes full cohort		B. Top 30 downregulated genes full cohort	
**Symbol**	**Log2 (FC)**	**Symbol**	**Log2 (FC)**
NPSR1	5.00481	MCOLN2	-3.70327
LYZ	4.73378	MT1L	-2.90424
RPS16	4.50878	FHL1	-2.58855
FBRSL1	3.73007	UGT2A3	-2.34656
PPDPF	3.61952	EPB41L3	-2.33001
MSLN	3.43814	RDX	-2.21112
FSTL1	3.2802	PTPRO	-2.09462
PHLDA2	3.15846	SPG20	-2.05226
PER1	3.1431	ZDHHC2	-1.93505
SLC28A2	3.07884	DRAIC	-1.91033
HSPB1	2.97515	NLRP2	-1.83767
ARHGDIA	2.95629	PRKAA2	-1.71988
ZYX	2.94151	SLC39A2	-1.70247
TACSTD2	2.85882	RPL9	-1.69293
CLDN3	2.8162	RETNLB	-1.68404
FLNA	2.76354	NFATC2IP	-1.61186
SCAND1	2.73961	LRP12	-1.59711
HIST2H3D	2.73145	B4GALNT2	-1.5582
APOBR	2.71831	ZNF43	-1.51893
BCL9L	2.66525	GABRA2	-1.45787
LTBP4	2.6512	HTR4	-1.45708
ARHGDIB	2.61455	URAD	-1.43137
RAVER1	2.61393	PHKA2	-1.42137
HOXB13	2.58295	NR1H4	-1.41512
ANPEP	2.58111	ANO5	-1.39797
SLC9A3R2	2.55819	RPS4Y1	-1.30354
LAMA5	2.55774	LPAR1	-1.29485
SOD3	2.54653	MIPOL1	-1.27782
PYY	2.539	DYNC1I1	-1.25938
NFIC	2.52708	EEF1A1P11	-1.25274
S100P	2.33816		

Supplementary Table 7: Gene enrichment for H3K27ac Colitis versus Non-IBD organoids*

Gene Symbol	Log2 (FC)
TSSC1	2.170274
PITX1	2.03683
STPG4	1.923237
B3GNTL1	1.881085
SDHA	1.80076
PDX1	1.789857
CCL28	1.676845
ABCA12	1.645783
APOL1	1.615867
FAM107B	1.57586
SULT1C4	1.57056
KPNA1	1.55623
AMN1	1.555893
MBOAT2	1.508358
CADPS2	1.504646
RAD51B	1.501779
FAM84A	1.476736
ZNF302	1.467879
BLVRA	1.453556
SMCO4	1.435216
LYZ	1.42346
HIVEP3	1.421301
NPSR1	1.420808
KCNJ3	1.416676
SEMA5A	1.408628
TBX15	1.4078
HSPBAP1	1.40598
SPICE1	1.400253
B3GALT5	1.391679
CDC42EP3	1.383068
WDR75	1.380746
SLC17A8	1.378241
HIVEP3	1.373505
SGMS1	1.362404
S100P	1.351097

*Represented top 35 upregulated genes comparing colitis to non-IBD.
